# Undetectable Free Aromatic Amino Acids in Nails of Breast Carcinoma: Biomarker Discovery by a Novel Metabolite Purification VTGE System

**DOI:** 10.3389/fonc.2020.00908

**Published:** 2020-06-30

**Authors:** Manmohan Mitruka, Charusheela R. Gore, Ajay Kumar, Sachin C. Sarode, Nilesh Kumar Sharma

**Affiliations:** ^1^Cancer and Translational Research Lab, Dr. D. Y. Patil Biotechnology & Bioinformatics Institute, Dr. D. Y. Patil Vidyapeeth, Pune, India; ^2^Department of Pathology, Dr. D. Y. Patil Medical College, Hospital and Research Centre, Dr. D. Y. Patil Vidyapeeth, Pune, India; ^3^Department of Oral Pathology and Microbiology, Dr. D. Y. Patil Dental College and Hospital, Dr. D. Y. Patil Vidyapeeth, Pune, India

**Keywords:** neoplasms, metabolic reprogramming, microenvironment, diet, free aromatic amino acids, biomarker

## Abstract

**Background:** Metabolic reprogramming in breast cancer is depicted as a crucial change in the tumor microenvironment. Besides the molecular understanding of metabolic heterogeneity, appreciable attention is drawn to characterizing metabolite profiles in tumor tissue and derived biological fluids and tissue materials. Several findings reported on the metabolic alterations of free aromatic amino acids (FAAAs) and other metabolites in biological fluids. Furthermore, there is a significant gap in the development of a suitable method for the purification and analysis of metabolite biomarkers in nails of cancer patients.

**Methods:** To address the metabolite alterations, specifically FAAA levels in nails, fingernail clippings of breast cancer patients (*N* = 10) and healthy subjects (N-12) were used for extraction and purification of metabolites. Here, we reported a novel and specifically designed vertical tube gel electrophoresis (VTGE) system that helped in the purification of metabolites in the range of 100–1,000 Da from nail materials. Here, the VTGE system uses 15% polyacrylamide under non-denaturing and non-reducing conditions, which makes eluted metabolites directly compatible with LC-HRMS and other analytical techniques. Qualitative and quantitative determination of FAAAs in nail lysates was done in positive ESI mode of the Agilent LC-HRMS platform.

**Results:** The analysis on collected data of nail metabolites clearly suggested that FAAAs including tryptophan, tyrosine, phenylalanine, and histidine were undetectable in nail lysates of breast cancer over healthy subjects. This is a first report that showed highly reduced levels of FAAAs in nails of breast cancer patients. Furthermore, the present observation is in consonance with previous findings that showed cancer cachexia and high amino acid catabolism in breast cancer patients that drive metabolite-led cancer growth and proliferation.

**Conclusion:** This paper provides a proof of concept for a novel and specifically developed VTGE process that showed first evidence on the undetectable level of FAAAs in nails of breast cancer patients as metabolite biomarkers. Here, the authors propose the potential use of a VTGE-assisted process to achieve metabolomic discovery in nails of breast cancer and other tumor types.

## Introduction

At the global level, it is estimated that the frequency of breast cancer among women is around 1.5 million each year. In the current decade, around 570,000 women have died from breast cancer, which is ~15% of all cancer deaths among women (1, 2). Besides the need for better efficacious drug options with precision-guided therapy, a need for the early diagnosis and monitoring of cancer drug treatment is at the forefront at preclinical and clinical levels (3, 4). To achieve the above goal, a search for new classes of biomarkers at the genome, transcriptome, small RNA, proteome, and metabolome level is highly relevant in a global context and Indian setting (5, 6).

Intra- and inter-tumor heterogeneity at both cellular and non-cellular levels is depicted as the origin of differential drug response in breast cancer (7–10). The elucidation of metabolic heterogeneity at tumor microenvironment and macroenvironment levels is important in the perspective of biomarker development and future interventions in breast cancer patients (11–16). Among metabolic heterogeneities, amino acid catabolism which targets free aromatic amino acids (FAAAs) such as tryptophan, tyrosine, phenylalanine, and histidine is regarded as one of the preferable hallmarks in breast cancer that support its growth and progression (17–22).

Determination of metabolite profiling was perceived as one viable option to detect early- and late-stage physiological and pathological changes in cancer patients (23–29). Several attempts were made in this direction by using metabolomic approaches such as LC-HRMS, GC-MS, and NMR spectroscopy. These metabolomic approaches were focused on the tumor tissue and biological fluids, including serum, urine, and saliva (30–36).

Recently, metabolomic studies in various biological fluids, including serum, urine, saliva, and tissues, are reported to highlight the importance of metabolite as a biomarker (15, 16, 23–29). Besides the use of traditional biological sources, the alternative use of nails as a source of biomarker discovery in breast cancer patients and other pathological conditions may have an added advantage over other biological fluids, precisely in terms of issues of contamination from microbes and environment compared to nails. Another interesting distinction is the following problem: the variability of metabolite concentration in biological fluids is related to the fasting/feed status of the donor, and at the same time, nails may show a stable concentration of metabolites. In the literature, limited attempts are available that investigated the nail metabolite profiling in environmental exposure cases and disease conditions (37–42). Interestingly, a metabolomic study of nail lysate in cancer patients has not been investigated previously. Till date, there is no suitable methodology that targets to profile nail metabolites of breast cancer and other tumor types.

In this paper, we report novel and specifically designed vertical tube gel electrophoresis (VTGE)-based metabolite identifications in nail lysates. Further, data reported that undetectable levels of FAAAs in nail lysate of breast cancer patients may serve as a potential diagnostic biomarker.

## Methods

### Study Population

Breast cancer patients (*N* = 10) and healthy women subjects (*N* = 12) were recruited from Dr. D. Y. Patil Medical College and Research Center, Pune, India. In accordance with the Institutional Ethics Committee (IEC), a formal approval was obtained to conduct research on healthy clinical subjects and breast cancer patients. The IEC is named as the Ethics Committee of Dr. D. Y. Patil Vidyapeeth, Pune, and this is affiliated to Dr. D. Y. Patil Vidyapeeth, Pune, India, with a registration no. Re-Reg.No.ECR/361/Inst/MH/2013/RR-16. The entire participating study population was apprised about the aims of the study, and informed consent was obtained prior to investigations.

Metabolite profiling data were analyzed between breast cancer patients and healthy subjects, without characterizing the malignancy into molecular subtypes and/or prior chemotherapy. The primary goals were to develop a protocol for the preparation of the nail metabolite lysate, followed by a purification process of nail metabolites and, finally, characterization with the help of LC-HRMS. At this stage of study, we focused on the novel methods and processes that helped to collect metabolite profiles in the nail lysates of breast cancer and healthy subjects. In future, the proposed novel methodology that uses VTGE and LC-HRMS technique may be used for a large study population of breast cancer patients with attributes of molecular subtypes and other clinical–pathological parameters.

### Preparation of Nail Lysates

Fingernail clippings were collected in a microfuge tube and cleaned to remove debris and environmental contaminations by using a mild detergent and 70% ethyl alcohol. Further, fingernail clippings were dried, weighed, and coded properly for healthy and breast cancer patients. Next, an equal amount of fingernail clippings (20 mg) was added to 800 μl of extraction buffer [Tris–HCl (20 mM, pH 8.5), 2.6 M thiourea, 5 M urea] and 800 μl beta-mercaptoethanol for lysis of fingernail clippings. The sample mixture was incubated for 24 h at 50°C under dark conditions and then centrifuged at 15,000 × RPM for 30 min. The supernatant was collected in a fresh microfuge tube and filtered by using a 0.45-micron syringe membrane filter. The above prepared nail lysates of healthy and breast cancer patients were diluted three times, stored, and labeled properly for purification by using the VTGE system.

### Purification of Metabolites by Using the VTGE System

In order to purify metabolites of nail materials, a novel and specifically designed VTGE system was used. It was standardized to use 15% polyacrylamide gel matrix to remove major components such as proteins, polysaccharides, large lipid molecules, and other various debris/contaminants. At the same time, this novel VTGE system allowed for the purification of metabolites in the range of 100 Da to 1,000 Da (43).

**Figure 1:**
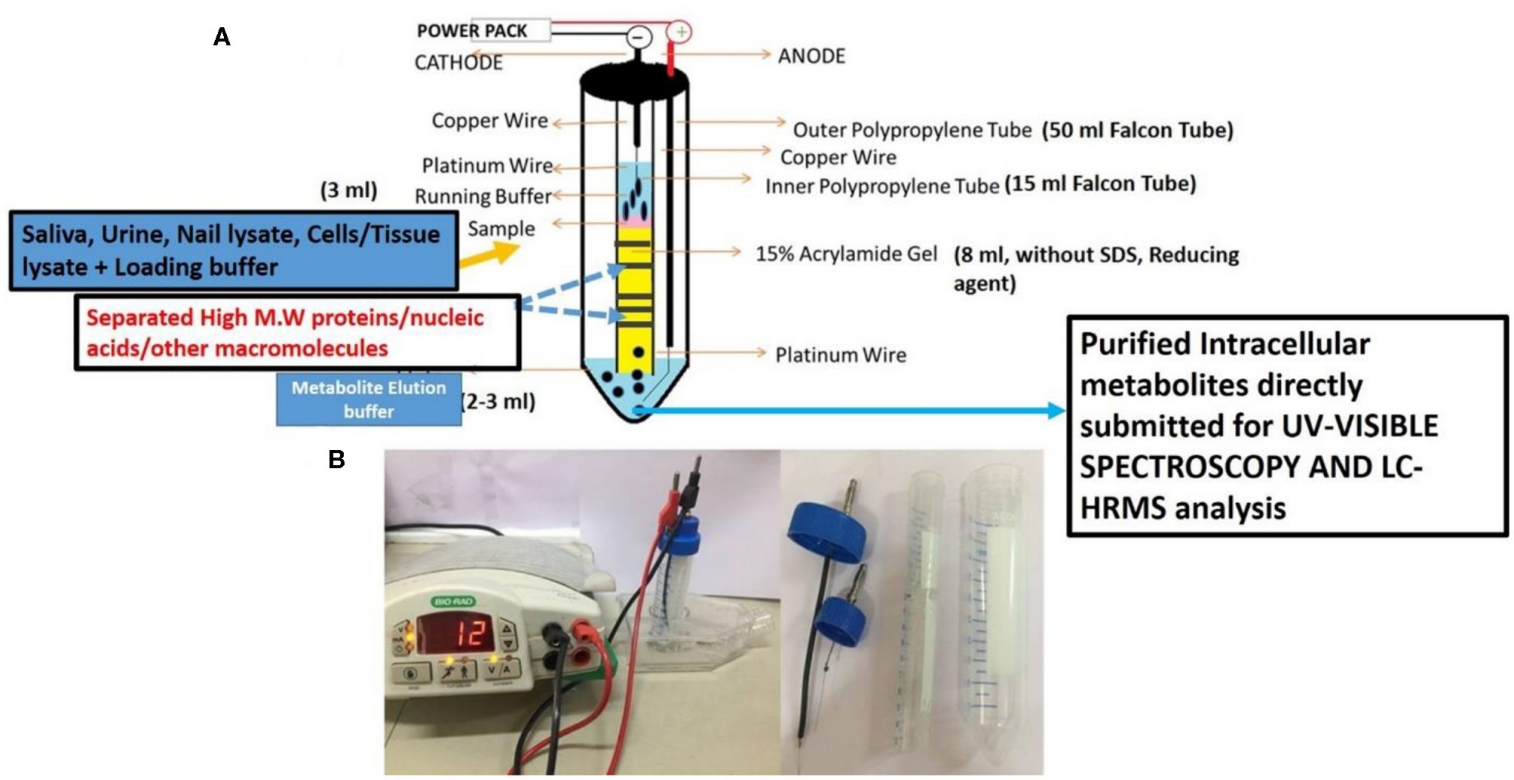
A flow diagram of a novel and specifically designed vertical tube gel electrophoresis (VTGE) system for intracellular nail metabolite purification. Here, **(A)** depicts the design, assembly, and key features including nature of matrix and non-reducing and non-denaturing buffers. **(B)** shows the working diagram that allows nail metabolite purification from healthy subjects and breast cancer patients.

The above prepared sterile and filtered nail lysate (750 μl) was mixed with 250 μl of loading buffer (4× glycerol and Tris pH 6.8) and electrophoresed on VTGE casted with 15% acrylamide gel (acrylamide: bisacrylamide, 30:1) as a matrix. The fractionated elute was collected in electrophoresis running buffer that contains water and glycine and excludes traditional SDS and other reducing agents. A notable distinctiveness of VTGE system is elaborated as regards the running buffer (3 ml) and elution buffer (3 ml) used, which are identical [water–glycine (192 mM), pH: 8.3]. A flow diagram of the VTGE method is presented in Figures 1A,B, which shows the assembly and design of the VTGE system. Nail lysate metabolites from healthy and breast cancer patients were eluted in the same running buffer, which is referred to as “elution buffer.” It was placed in the lower electrophoretic tube that has an anode wire. The voltage and current ratio from the power supply was maintained to generate 1,500–2,500 mW to achieve the electrophoresis of biological samples. The total run time allowed was 2 h, after which the sample was collected in a fresh microfuge tube for direct LC-HRMS characterization. Interestingly, the pH of the eluted metabolite buffer was measured for healthy and breast cancer patients which ranged between 3.0 and 3.5 acidic pH. An acidic pH (2.5–4.0) buffer containing metabolites is known to increase the ionization efficiency during LC-HRMS analysis (44). At the end of the run, the inner tube containing polyacrylamide gel was removed and placed for Coomassie Brilliant Blue dye staining to ensure that protein components of nail lysates were trapped in the polyacrylamide matrix. Eluted nail metabolites from healthy and breast cancer patients were stored at −20°C for further direct identification by the LC-HRMS technique.

### Identification of Potential Nail Metabolites by LC-HRMS

The purified nail metabolites from the VTGE system were submitted for LC-HRMS analysis. For the liquid chromatography (LC) component, the RP-C18 column used was Zorbax, 2.1 × 50 mm, 1.8 μm. Further, at a flow rate of 0.2 ml/min, a gradient was formed by mixing mobile phases A (water containing 5 mM ammonium acetate) and B (0.2% formic acid). For running the sample, an injection volume of 25 μl and a flow rate of solvent maintained at 0.3 ml per minute were used. The HPLC column effluent was allowed to move using an electrospray ionization triple quadrupole mass spectrometer (Agilent Technologies). For mass identification of nail metabolites, samples were run in positive electrospray ionization (ESI) M–H mode. During LC-HRMS analysis, the mass spectrometer component used was MS Q-TOF quadrupole time-of-flight mass spectrometer (Q-TOF-MS) (Agilent Technologies, 6,500 Series Q-TOF LC/MS System) in dual AJS electrospray ionization (ESI) mode. The acquisition mode of MS1 was recorded with a minimum value of m/z at 60 and a maximum value of m/z at 1,700.

## Statistical Analysis

Data are presented as the mean ± SD. The statistical significance between the healthy subjects and breast cancer patients was assessed with the help of one-way ANOVA test. Data calculation and statistical tests were performed by SPSS version 15.0 (SPSS) software package.

## Results and Discussion

The importance of metabolite adaptations and profiling of metabolites in fine-needle aspiration biopsies and surgical biopsies using serum, urine, and saliva was shown in various tumor types such as breast, liver, thyroid, and colorectal cancer (31–36). However, it is important to note that metabolite profiling in nail materials of cancer patients, including breast cancer patients, has not been reported in literature. Moreover, a suitable method and process to profile metabolites from a nail lysate of other human disease is completely lacking in the field of science.

### Novelty of the VTGE Process

To achieve new and additional knowledge on metabolomic biomarkers in breast cancer, we developed a novel and specifically designed VTGE system that involved methods to purify nail lysate metabolites. The lysates are directly compatible with LC-HRMS techniques without any further need of complicated extractions, modifications, and labeling protocols to identify and estimate metabolite biomarkers. Among a pool of various metabolites, FAAAs are considered as key tumor metabolites that support growth and progression (17–22, 30). However, there is not a single paper that addresses the importance of FAAAs as potential biomarkers in nail materials of breast cancer patients. To address the need of non-invasive, cheaper, and rapid methods for the metabolite biomarkers in breast cancer, the authors present a novel and specifically designed VTGE-based purification of metabolites (100–1,000 Da) in nails of breast cancer patients. In this paper, the reported VTGE method is simply an out-of-box innovative application with respect to the classical (45) system that is primarily used for the separation and purification of large macromolecules such as proteins and nucleic acids. In this novel method, the authors have designed a VTGE system with the help of laboratory plasticware that purifies nail metabolites (100 Da−1,000 Da) by direct elution in the lower running buffer.

### Nails Accumulate Metabolites

Limited reports support the avenues to explore nail metabolites, including ethyl glucuronide in the keratinous matrices of nail materials of patients (38–42). It is worth mentioning that glucuronide derivatives are hallmarks of liver metabolism for drug treatment. Therefore, the presence of ibuprofen glucuronide in nails of healthy and breast cancer patients is supported by previous reports on detection of ethyl glucuronide in keratinous matrices of nails. Furthermore, the reported novel VTGE system-assisted nail metabolite profiling approach is convincing and in line with existing but highly limited reports. Furthermore, acid digestion and SPE cleanup of metabolites derived from alternative plasticizers and flame retardants such as bis(2-propylheptyl) phthalate (DPHP), bis(2-ethylhexyl) terephthalate (DEHTP), bis(2-ethylhexyl) adipate (DEHA), and 2,2-bis (chloromethyl)-propane-1,3-diyltetrakis(2-chloroethyl) bisphosphate (V6) in nail materials of selected participants (38, 40). A nail metabolite profiling study reported the use of ball mill grounding and extraction process followed by UHPLC-triple quadrupole-mass analysis (39).

**Figure 2:**
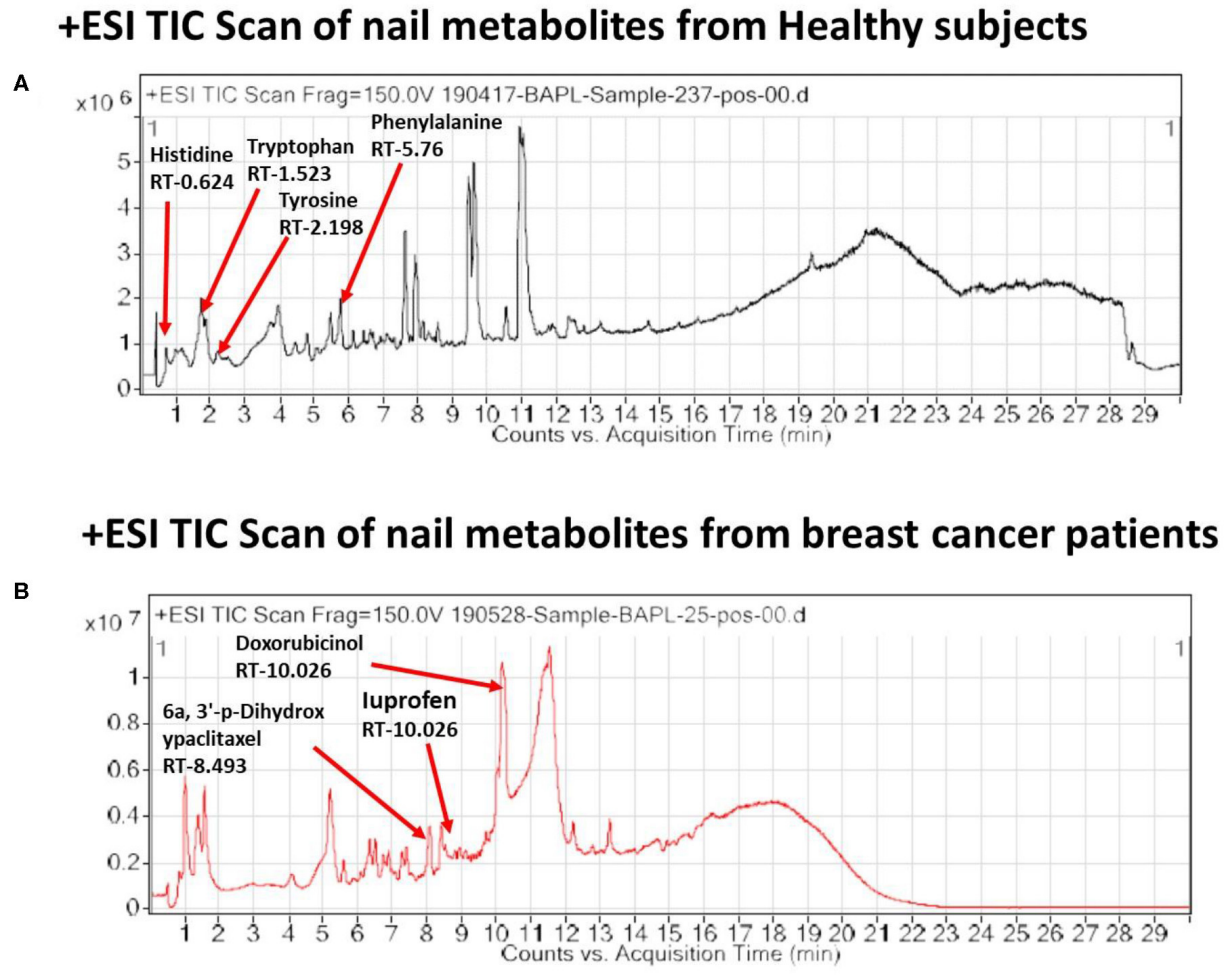
VTGE-purified nail metabolites identified by LC-HRMS show unique TIC in healthy subjects over breast cancer patients. To identify and profile nail metabolite changes, VTGE system-assisted purified nail metabolites were directly submitted to LC-HRMS with positive ESI mode. Total ion chromatogram (TIC) of purified nail lysate of healthy subjects and breast cancer patients is given in **(A)** and **(B)**, respectively.

In the light of negligible approaches to profile nail metabolites in breast cancer and other disease conditions, we suggest that the VTGE system-assisted purification process with the LC-HRMS technique is novel and highly efficient. The data clearly showed that the identified nail metabolites, including FAAAs and drug metabolites, were detected in positive ESI total ion chromatogram of healthy subjects (Figure 2A) that showed tryptophan (RT-1.523), L-phenylalanine (RT-5.76), L-tyrosine (RT-2.198), and histidine (RT-0.624). On the other hand, positive ESI total ion chromatogram of breast cancer patients (Figure 2B) indicates presence of anti-cancer drug metabolites, namely, 6a,3′-p-dihydroxypaclitaxel (RT-8.493) and doxorubicinol (RT-10.026). In fact, ibuprofen glucuronide (RT-8.607), a well-known metabolite of anti-inflammatory drug ibuprofen, was detected in both healthy subjects and breast cancer patients. This clearly shows the precision and efficacy of VTGE-assisted nail metabolite purification and identifications by LC-HRMS. Finally, positive ESI chromatogram reveals undetectable FAAA metabolites in nails of breast cancer patients compared to highly abundant in the case of healthy subjects.

### Identifications of Drug Metabolites in Nail

**Figure 3:**
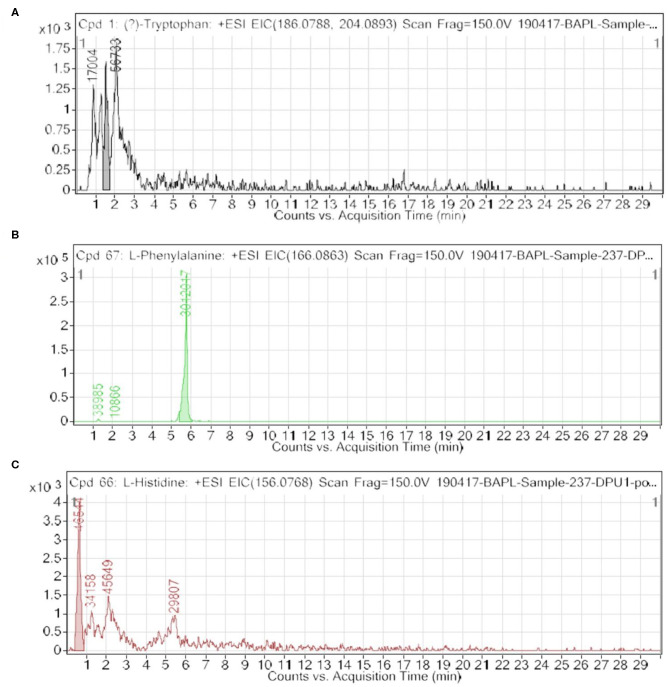
An extracted ion chromatogram (EIC) of drug-metabolized products in nail lysates; a first report is distinctively detected in healthy subjects and breast cancer patients. Here, **(A–C)** represent a +ESI EIC of ibuprofen glucuronide (+ESI EIC 364.1517, 382.1622), 6a,3′-p-dihydroxypaclitaxel (+ESI EIC 871.2682, 872.2716, 889.2788, 890.2821), and doxorubicinol (+ESI EIC 527.1786, 528.1819, 545.1892, 546.1925), respectively.

Furthermore, we looked into the LC-HRMS-identified metabolites other than FAAAs and the data suggest that various kinds of drugs including anti-inflammatory, anti-analgesic, antibiotics, and anti-cancer drugs are actually accumulated in the nails of selected clinical subjects. Some of notable anticancer drug metabolites such as 6a,3′-p-dihydroxypaclitaxel and doxorubicinol were exclusively highly abundant in nail lysates of most of selected cancer patients. A positive ESI extracted ionization chromatogram (EIC) is presented as 6a,3′-p-dihydroxypaclitaxel (+ESI EIC 871.2682, 872.2716, 889.2788, 890.2821) and doxorubicinol (+ESI EIC 527.1786, 528.1819, 545.1892, 546.1925) in Figures 3B,C, respectively. Furthermore, a positive ESI EIC of ibuprofen glucuronide (+ESI EIC 364.1517, 382.1622), a well-known anti-inflammatory drug, was identified in both healthy subjects and breast cancer patients (Figure 3A, Table 1B). Parameters such as abundance, mass value, and m/z value of identified drug metabolites in nails of selected healthy and breast cancer patients are given in Tables 1, 2. On the one hand, anti-inflammatory drug metabolites such as ibuprofen glucuronide are detected in both healthy and cancer patients. This observation strongly supports the precision and reproducibility of the VTGE process for the improved precision and efficacy during LC-HRMS metabolite identifications. Besides the above identified FAAAs and drug metabolites, VTGE-assisted purified nail metabolites revealed the presence of smallest-size metabolite 3-methylbut-3-enoic acid with a m/z value of 101.6 and a mass value of 100.0524 Da. On the other hand, the highest mass metabolite detected in nails was 28-glucosylarjunolate 3-[rhamnosyl-(1->3)-glucuronide] with an m/z value of 973.4944 and a mass value of 973.4944 Da. Here, the authors would like to draw attention on these two metabolites being known to be secreted in urine. Therefore, findings collected by VTGE assisted nail metabolite purifications are further strengthened. Interestingly, the mass range of identified nail metabolites matches with this novel VTGE-assisted purification approach and this encouraged us to explore the distinct nail metabolite profiling between healthy and breast cancer patients. Here, the authors point out that detection of these unique metabolized products and drug metabolites in the nail lysates of healthy and breast cancer patients is a first report in the literature. This brings evidence that the presence of drugs and their metabolites corroborates a chance of success with reference to the highly distinct abundance of FAAAs in healthy subjects over breast cancer patients.

**Table 1A:** List of free aromatic amino acids and their abundance in nail lysates.

**Sr. no**	**Name of free aromatic amino acids**	Healthy subjects^*^	**Breast cancer patients**
1.	L/D-Tryptophan	18407 ± 5071	Undetectable
2.	L-Phenylalanine	3012017 ± 80100	Undetectable
3.	L-Tyrosine	193292 ± 40817	Undetectable
4.	Histidine	46544 ± 8981	Undetectable

* *Data represent mean ± S.D (N = 10 breast cancer patients and N = 12 for healthy subjects)*.

**Table 1B:** List of drugs and derived metabolites and their abundance in nail lysates.

**Sr. no**	**Name of free aromatic amino acids**	**Healthy subjects**	**Breast cancer patients**
1.	6a,3′-p-Dihydroxypaclitaxel	Undetectable	11950 ± 6625^*^
2.	Doxorubicinol	Detectable	143966 ± 56727^*^
3.	Ibuprofen glucuronide	13546 ± 9872^*^	11956 ± 4241^*^

* *Data represent mean ± S.D (N = 10 breast cancer patients and N = 12 for healthy subjects)*.

**Table 2:** List of free aromatic amino acids and their abundance in nail lysates.

**Sr. no**	**Name of nail metabolite FAAAs and drugs metabolite**	**Formula**	**RT**	**m/z**	**Mass**	**Polarity**
1	(?)-Tryptophan	C11 H12 N2 O2	1.523	186.0772	204.0894	Positive
2	L-Phenylalanine	C9 H11 N O2	5.76	166.0866	165.0793	Positive
3	L-Tyrosine	C9 H11 N O3	2.198	182.0813	181.0739	Positive
4	Histidine	C6 H9 N3 O2	0.624	156.0756	155.0682	Positive
5	6a,3′-p-Dihydroxypaclitaxel	C45 H47 N O18	8.493	889.2772	889.2776	Positive
6	Doxorubicinol	C27 H31 N O11	10.026	545.1857	545.1869	Positive
7	Ibuprofen glucuronide	C19 H26 O8	1.981	382.1627	382.1619	Positive

### Reduction in the Level of FAAAs in Breast Cancer

**Figure 4:**
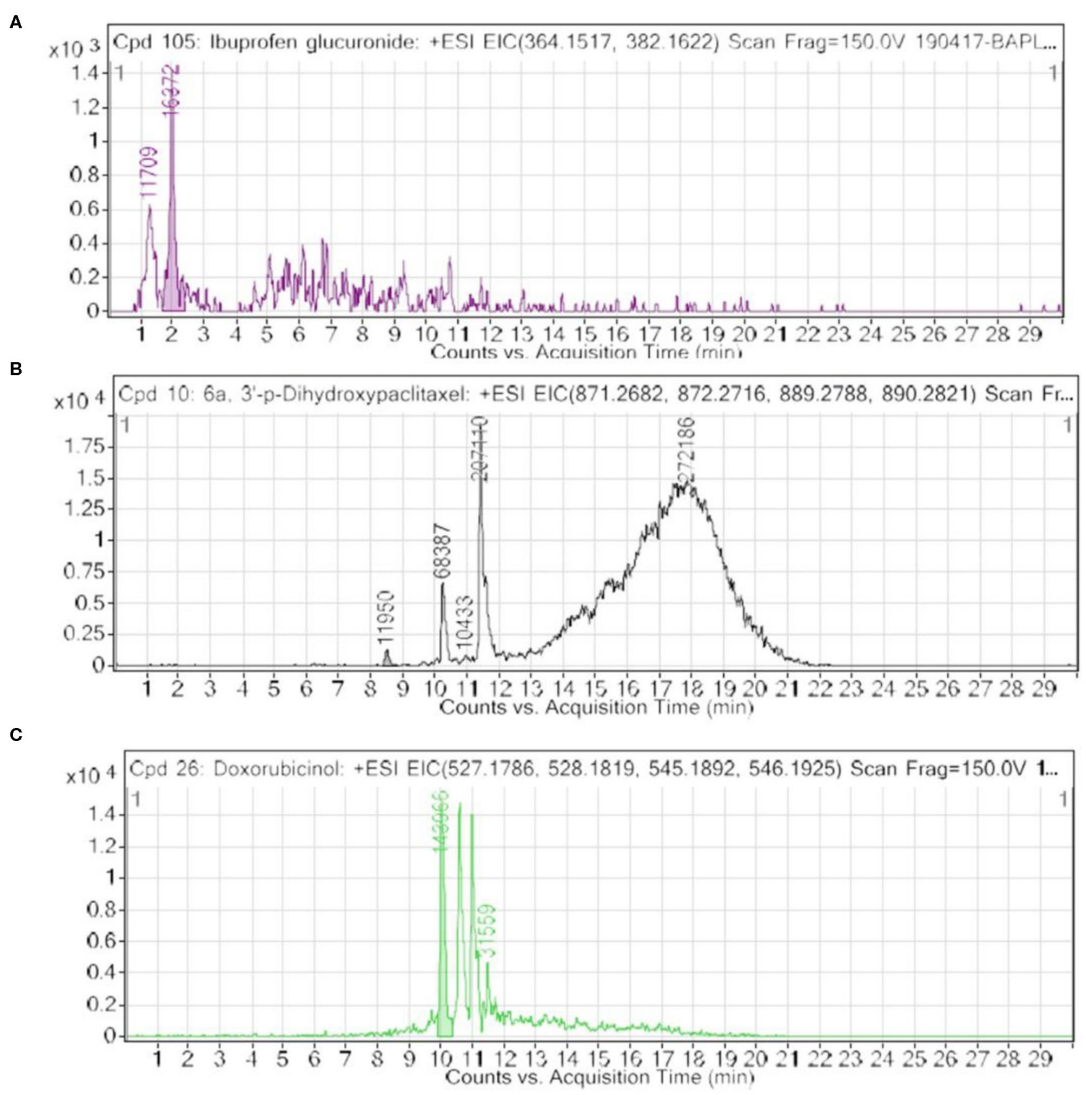
An extracted ion chromatogram (EIC) of free aromatic amino acids (FAAAs) in nail lysates; a novel report shows high abundance in healthy subjects and undetectable in breast cancer patients. Here, **(A–C)** represent a +ESI EIC of tryptophan (+ESI EIC 186.0788, 204.0893), phenylalanine (+ESI EIC 166.0863), and histidine (+ESI EIC 156.0768), respectively.

Among the key roles of amino acid metabolism in the tumor microenvironment, the role of tryptophan and catabolism of other aromatic amino acids is suggested to support an immunosuppressive landscape (17–22, 30–32). In essence, amino acid catabolism including tryptophan plays an important role in immune heterogeneity in the tumor niche. The metabolized product of tryptophan, kynurenine, is suggested to promote differentiation of T regulatory cells, which helps in immunosuppression. At the molecular level, either of the two enzymes indoleamine-2,3-dioxygenase (IDO-1/-2) and tryptophan 2,3-dioxygenase 2 (TDO2) is known to initiate catabolism of tryptophan, which leads to the generation of l-kynurenine (21, 22, 36). In the present paper, we showed clear and novel evidence on the appreciable level of FAAAs including tryptophan, phenylalanine, tyrosine, and histidine in the nail lysate of healthy subjects (Table 1A). Interestingly, among FAAAs, phenylalanine was found to be highest in abundance, followed by tyrosine, histidine, and tryptophan. On the other hand, the level of FAAAs was undetectable in the nail lysate of breast cancer patients. Here, the reported methodology is suggested to reveal undetectable levels of FAAAs in the nail lysate of breast cancer patients as potential metabolite biomarkers over healthy subjects (Figure 4, Tables 1, 2). A representative positive EIC of tryptophan (+ESI EIC 186.0788, 204.0893), phenylalanine (+ESI EIC 166.0863), and histidine (+ESI EIC 156.0768) is illustrated in Figures 4A–C, respectively. The TIC and EIC of identified FAAAs in the nails of healthy subjects as well as those undetectable in breast cancer patients are vividly determined quantitatively by LC-HRMS techniques. The highly efficient mass ionization of FAAAs identified in nails is credited to the novel purification methods assisted by the VTGE system. Indeed, this observation is in consonance with the existing views of pro-tumoral effects which attributed to altered amino acid catabolism and their derived products.

Metabolic dependency and addiction to amino acids by cancer cells are known to support self-sustained growth and aggressiveness (23–29). In the case of breast cancer patients, abundance of key amino acids, including FAAA in the other body parts and biological fluids, is reduced. This metabolic adaptation in breast cancer patients may be the factor behind undetectable levels of FAAAs as compared to healthy subjects. At the same time, availability of FAAA in the tumor mass is elevated and the catabolized products of these amino acids are increased (6, 26–29). A metabolomic study on pancreatic and ovarian cancer indicates that glutamine citrulline and histidine can serve as potential biomarkers (25, 29). In prostate, colorectal, and breast cancer patients, the levels of neopterin- and tryptophan-metabolized metabolites in serum are suggested to be predictive and prognostic factors (21, 30).

The reduction of FAAAs in the nail lysate of breast cancer patients compared to healthy subjects is in accordance with the recent study that showed low levels of serum-free amino acids, including arginine, alanine, isoleucine, tyrosine, and tryptophan in breast cancer (stages I–III) patients (33). Moreover, a liquid chromatography-tandem mass spectrometry (LC-MS/MS) metabolic profiling and bioinformatics analysis showed alterations in arginine/proline metabolism, tryptophan metabolism, and fatty acid biosynthesis in breast cancer patients (26).

**Figure 5:**
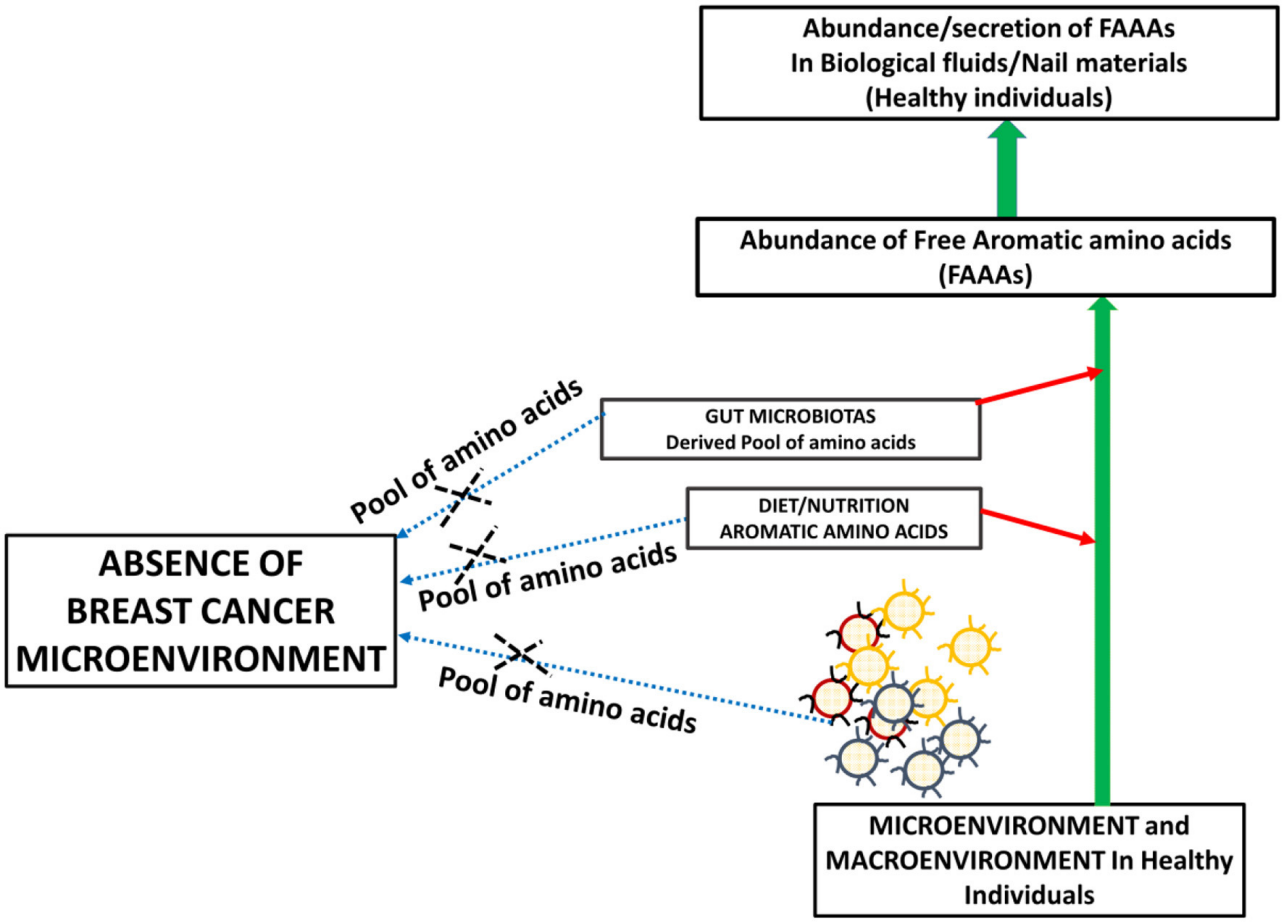
A proposed model supporting the high abundance of FAAAs in biological fluids and tissue materials including nails of healthy subjects.

**Figure 6:**
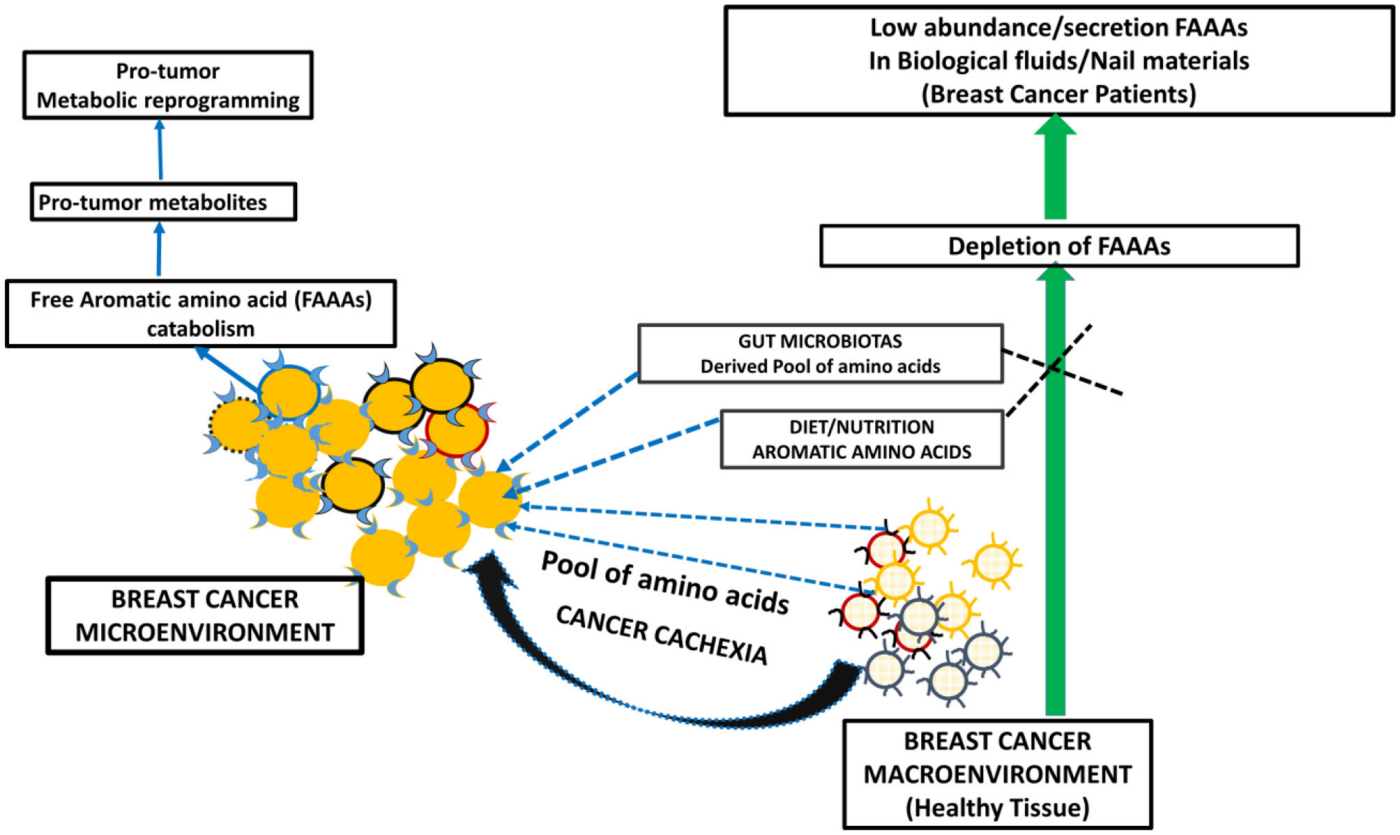
A proposed model of metabolic reprogramming in breast cancer microenvironment and macroenvironment, which leads to FAAA catabolism and in turn undetectable level of FAAAs in nails and other non-tumor biological fluids and materials.

Metabolite profiling suggests tyrosine, phenylalanine, or tryptophan decrease in the serum of patients with gastroesophageal cancer. At the same time, these levels are higher in tumor tissue of gastroesophageal cancer (20). The bioavailability of plasma-free amino acids is implicated in alerting the tumor protein synthesis, which is widely considered as one of the tumor hallmarks. In fact, the profile of plasma-free amino acids, especially aromatic amino acids tyrosine, phenylalanine, tryptophan, and histidine, is changed according to the stages and types of cancer. In another way, intra- and inter-tumor heterogeneity may play a role in the distinct profile of free aromatic amino acids (18). Furthermore, a NMR spectroscopy analysis of plasma samples of brain tumor patients showed the elevated glycolytic pathway and low abundance of glutamine (24). Existing views on amino acid catabolism as a hallmark of breast and other cancer types are supported by additional and crucial evidence that supports that breast cancer patients have undetected levels of FAAAs in nails compared to healthy subjects (Figures 5, 6). Wherein, the accumulation of FAAAs in nails of healthy subjects should be viewed in line with the metabolic landscape during normal physiological conditions, which also holds true for other biological fluids.

## Conclusion

Despite emanating understanding on genetic, epigenetic, and environmental factors that impact tumorigenesis, a role of metabolic reprogramming is highly appreciated in tumor heterogeneity. A better understanding about metabolic reprogramming is crucial for development of prognostic markers, diagnostic avenues, therapeutic monitoring, and combinatorial drug therapy. In the present paper, use of a metabolomic approach to characterize metabolites qualitatively and quantitatively in various tissue samples and non-invasive biological fluids/materials including nails is emphasized at preclinical and clinical levels. The authors propose the use of this novel VTGE system-based purification of nail metabolites and their precise estimation by LC-HRMS for future potential as cancer biomarkers. It is the first of its kind and a novel study on breast cancer metabolomic in nail lysate. Furthermore, the described methods and processes warranted future studies to establish metabolite biomarkers in other biological fluids/tissues in various cancer models. Future studies should also focus on the level of FAAAs in nails of healthy subjects and breast cancer patients as a clinical assay in the future.

## Data Availability Statement

The datasets generated for this study are available on request to the corresponding author.

## Ethics Statement

The studies involving human participants were reviewed and approved by the Institutional Ethics Committee (IEC) of Dr. D. Y. Patil Vidyapeeth, Pune, India. The patients/participants provided their written informed consent to participate in this study.

## Author Contributions

MM: collection of data. CG: clinical data. AK: collection of data. SS: preparation of manuscript and editing. NS: conceive of idea, methodology, preparation of manuscript, and editing. All authors contributed to the article and approved the submitted version.

## Conflict of Interest

The authors declare that the research was conducted in the absence of any commercial or financial relationships that could be construed as a potential conflict of interest.
